# Work design, employee well-being, and retention intention: A case study of China's young workforce

**DOI:** 10.1016/j.heliyon.2023.e15742

**Published:** 2023-04-24

**Authors:** Xuelin Chen, Abdullah Al Mamun, Mohammad Enamul Hoque, Wan Mohd Hirwani Wan Hussain, Qing Yang

**Affiliations:** a School of Business, Jishou University, 416000 Jishou City, Hunan, China; b UKM - Graduate School of Business, Universiti Kebangsaan Malaysia, 43600, UKM Bangi, Selangor, Malaysia; c BRAC Business School, BRAC University, Dhaka 1212, Bangladesh

**Keywords:** Work design, Retention intention, Job characteristics, Work relationship, Work condition

## Abstract

China's growing workforce of young employees has propelled its economy towards becoming a global power. However, with evolving workplace difficulties and uncertainties, the rate of employee turnover is also rising, which affects every department in companies, in addition to impacting costs and finances. This study explored the influences of five core job characteristics, work relationships, and work conditions on young Chinese employees' retention intentions, mediated by employee well-being. Using a quantitative cross-sectional approach, 804 responses were obtained from young Chinese workers. We also employed partial least squares structural equation modeling to analyze and forecast the extent of the impact of this study's independent variables. The empirical findings revealed that job autonomy, skill variety, task significance, feedback, work relationships, and work conditions indirectly influenced the retention intentions of young workers in China, with employee well-being acting as a mediator. However, the impact of task identity on employee well-being and retention intentions was insignificant. Our study contributes to the literature on employee retention intentions by demonstrating the importance of young employees' perceptions of work design-related aspects and extending the application of the job characteristics model.

## 1 Introduction

Continuous changes in the global economy have increased work- and career-related uncertainty, leading to high labor mobility, restructuring, and transformation of the workplace. Hence, firms face unprecedented challenges in the engagement and retention of young employees [1]. Therefore, organizations must take notice of young employees because they are deemed productive in the digital age and can multitask [2]. However, studies have shown that young employees get more easily overwhelmed, resulting in reduced long-term commitment to companies and a greater tendency to quit when conditions are not ideal [3]. High retention intention (RI) among employees indicates a greater likelihood of remaining with their current employer, leading to an increased contribution to the organization's growth and success. Conversely, low RI increases the risk of employee turnover, with potentially negative consequences for organizations such as the loss of experienced staff, decreased productivity, lower morale, and increased recruitment and training costs [[4], [5], [6]]. With China transitioning from a manufacturing-based economy to a more service-oriented economy, young workers are becoming increasingly important in driving the country's growth and development. Therefore, finding ways to increase young workers' RI is vital. In this regard, greater knowledge of the nature of work can help improve how companies design work for young employees.

China is the most populous country in the world, with the largest share of young workers. The China State Council categorizes young employees as those aged between 14 and 35 years [7]. Based on a 2020 census survey, there are approximately 400 million youths, accounting for around 28.4% of the total population and 31.6% of the total workforce in China [8]. The youth unemployment rate is expected to be nearly 20% in 2022 [9], and the condition can worsen as approximately 10.76 million Chinese students are expected to graduate by 2022 [10]. A 2018 survey conducted by the China Youth Daily collected data from 1972 employees aged between 10 and 35 and showed that around 23% of young workers experienced naked resignation (quitting one's job before finding a new one), and nearly half considered it but never did so [11]. This finding strongly indicates that only a small percentage of young employees intend to continue working at their current workplaces. The shrinking labor force caused by China's aging population further highlights the urgency of retaining young workers [12]. Thus, comprehending the factors that influence RI among young workers is paramount for organizations that aim to retain this young demographic and harness its potential.

Young workers have several unique characteristics. Previous studies have pointed out that young workers have an identity independent from that of their organizations and are less loyal towards their organizations than older workers [13,14]. They are particularly concerned about their personal feelings at work and place a high value on employee well-being (EW) [15]. In fact, the development of EW could be a positive intervention in reducing workers' intention to leave their jobs [16]. It is rational to assume that once young employees are convinced that aspects related to their organization's work design are unsatisfactory, their EW is likely to decrease, which would consequently lower their RIs compared with older employees. Numerous studies have explored the factors that influence RI [[17], [18], [19]]. However, research specifically focusing on young workers' RIs in the context of their work design is scarce.

Therefore, to fill this research gap, this study investigates the impact of factors such as work design, work relationships (WRs), and work conditions (WCs) on young Chinese employees' RIs, with employee well-being (EW) serving as a mediator in this relationship. This study seeks to understand how young Chinese workers' perceptions of work design influence their RIs. In doing so, this study combines the job characteristics model (JCM) and the job demand-resources (JD-R) model to determine the associations among job characteristics, WRs, WCs, EW, and RI. The JCM and JD-R models offer well-established, comprehensive, and practically relevant frameworks for understanding young Chinese employees’ RI. These two theories have been tested and validated in various cultural contexts, including China. Their applicability to the Chinese context suggests that they may provide useful insights for organizations seeking to retain young employees [20,21]. Previous research on JCM has shown that there are five core job characteristics that can induce positive job attitudes (i.e., RI) among employees, that is, job autonomy (JA), skill variety (SV), task identity (TI), task significance (TS), and feedback (FB) [21,22]. However, these studies have not examined how job characteristics influence RI. Moreover, it remains unclear whether this effect is direct or indirect. The JD-R model is more suitable for addressing this problem because it considers WRs and comfortable WCs as job resources that have a positive effect on employee work attitudes and behaviors [20,23]. The JD-R model is more comprehensive and includes several determinants. It is also more adaptable and can be customized for a wide range of work settings [23].

Based on this discussion, a conceptual framework was developed and empirically tested among young Chinese workers. This study explores the mechanism by which the core job characteristics, WR and WC, influence the RI of young workers in China, given that these factors are particularly relevant for young Chinese workers, who have unique expectations and work values compared to other demographics [24]. In this way, the research can provide a more targeted and detailed analysis of the specific factors that are most important for predicting RI among young Chinese workers. They can also provide more precise and actionable information for organizations seeking to improve retention rates. The study's findings contribute in the following ways: First, by investigating whether the influence of five job characteristics, WR, and WC, on RI is direct or indirect through the mediator of EW, this study will clarify the relationships among these variables. Previous research has indicated that these job-related variables are associated with RI; however, the nature of this relationship remains unclear. This study contributes to the understanding of the mechanisms by which these variables affect RI. Second, it highlighted the importance of EW as a mediator between job-related factors and RI. Earlier studies have shown that EW is associated with positive outcomes such as job satisfaction, organizational commitment, and productivity [25,26]. This study addresses this research gap by demonstrating that EW is a key factor in determining RI. This study adds to the existing literature by demonstrating the complex interactions among different job-related factors, EW, and RI. This will provide a more nuanced understanding of the factors influencing RI, while emphasizing the need for a holistic approach to address employee retention issues.

## 2 Literature review

### 2.1 Theoretical background

#### 2.1.1 Job characteristics model

The JCM is a widely accepted model for job redesign. To better understand RI, this study used the JCM as a suitable and appropriate theoretical model to examine the factors influencing employee RI in the context of work design. The JCM argues that enriching or complex jobs are associated with increased job satisfaction, motivation, and work performance [27]. More specifically, they assumed that five core job characteristics (i.e., JA, SV, TI, TS, and FB) affect employees' critical psychological states, which, in turn, enhance their work attitudes, such as work engagement and RI. Furthermore, the five core job characteristics of the JCM can be combined into a single index of motivating potential score (MPS), which reflects the overall potential of a job to influence an individual's feelings, intentions, and behaviors [21].

#### 2.1.2 JD-R model

As an extension of the JCM, the JD-R model has gained popularity among researchers, guiding the understanding of specific workplace characteristics that can lead to positive and negative organizational outcomes [28]. The JD-R model presents two sets of variables that can be identified in any job setting: job demands and resources [29]. Job resources refer to the physical, social, and organizational resources provided by a job to individual employees. Compared with job resources, job demands refer to the physical, psychological, social, and organizational aspects of a job that require continuous physical, cognitive, or emotional efforts and may cause stress [23]. Schaufeli and Bakker [30] presented a revised version of the JD-R model and considered burnout and well-being as mediators of the relationship between job demands and negative outcomes (e.g., health problems) and job resources and positive outcomes (e.g., RI).

### 2.2 Hypothesis development

#### 2.2.1 Job characteristics and employee wellbeing (EW)

Work design has a significant influence on organizational success and individual well-being [31]. Hackman and Oldham [32] suggested that well-designed jobs have five characteristics that define their motivating potential: JA, SV, TS, TI, and FB. Motivational job characteristics are the key job resources that stimulate EW [30]. According to Hackman and Oldham [27], these characteristics enhance job attitudes by shaping three critical psychological states: job meaningfulness, a sense of responsibility for work outcomes, and knowledge of job outcomes. This study adds to the JCM by considering the different psychological states of EW. According to Parkes [33], EW refers to the overall quality of an employee's experience and functioning at work. Employees function effectively when they experience satisfaction and positive situations in the workplace, which enhance their level of well-being [34].

Motivational job characteristics are key resources that stimulate work engagement [30]. Wan et al. [22] stated that each job has a specific motivational potential to foster work engagement, which is defined as a positive work-related state of mind characterized by feelings of vigor, dedication, and absorption. This finding suggests a positive relationship between five core job characteristics and EW. According to Kasser [35], by promoting the satisfaction of the four psychological needs of security, competence, relatedness, and autonomy, individuals' well-being can be higher. Carvalho and Chambel [36] suggested that employees with elevated levels of job autonomy are more effective at balancing their professional and personal lives, leading to a heightened perception of their work as meaningful. Krasman [37] discovered that skill variety, which refers to the extent to which a job necessitates diverse activities and utilizes the various abilities and competencies of the employee, significantly influences favorable employee attitudes and behavioral outcomes. Furthermore, task significance reflects the degree to which a job influences the lives or work of others, whether inside or outside an organization [32]. As Hackman and Oldham [27] asserted, individuals in occupations that considerably influence others’ physical or psychological well-being are more likely to perceive their work as meaningful. In addition, Hackman and Oldham [27] illustrated that jobs that involve an entire task, such as providing a complete unit of service or assembling an entire product, are always more interesting to perform than those that involve only small parts of the task. In other words, jobs where workers can form a task identity (TI) are invariably more meaningful than jobs with only incomplete tasks. Based on the aforementioned, it is reasonable to infer that job positions possess five job characteristics that motivate individuals to enhance their work experience and overall functioning. Johari et al. [34] proposed that the psychological meaningfulness of a job is determined by a combination of these five core job characteristics.

H1 JA positively influences EW

H2 SV positively influences EW

H3 TI positively influences EW

H4 TS positively influences EW

H5 FB positively influences EW

#### 2.2.2 Work relationship (WR) and EW

With the growing global concern for employee well-being in organizations, more attention should be paid to the influence of WRs [38]. Sias et al. [39] defined WR as “any relationship one has with a coworker, such as a supervisor-subordinate, peer, or mentoring relationship.” Numerous researchers have studied the WRs between supervisors and employees as well as those among employees to find correlations with employee work attitudes [41]. Epitropaki et al. [40] highlighted that high-quality (and relatively higher) WRs in the workplace benefit employees, making them more satisfied with their work. According to Hackney et al. [41], supervisor–subordinate WR quality neutralizes negative attitudinal and behavioral strains in employees. In line with the JD-R model, the availability of various job supports can potentially activate a motivational pathway that satisfies employees' fundamental needs for growth and development. This pathway facilitates the achievement of work-related goals, leading to an increase in individuals’ overall satisfaction with their work environments [42]. High-quality WR, as a job resource, is likely to enhance EW. Hence, we propose the following hypothesis:

H6 WRs positively influence EW

#### 2.2.3 Work condition (WC) and EW

WCs reflect the environment in which a job is performed [43]. WCs include health hazards [44] and noise, temperature, and cleanliness of the working environment [43,45]. The condition of the work environment significantly impacts an individual's work attitude and is deemed favorable or appropriate when conducive to optimal, healthy, safe, and comfortable work practices [46]. Employees are more likely to perform at their optimal level when WC is comfortable and supportive because such an environment tends to foster higher levels of employee satisfaction [47]. Poor workplaces are the fundamental reasons for poor well-being and low staff RIs [48]. According to the JD-R model, EW plays a key role in the association between job resources and positive outcomes [30]. As a job resource, supportive WC may enhance EW because of its intrinsic and extrinsic motivational qualities, in turn facilitating positive job outcomes such as low turnover intention [22]. It is rational to assume that the quality of WCs influences EW. Hence, we propose the following hypothesis:

H7 WCs positively influence EW

#### 2.2.4 EW and RI

According to recent managerial practices, EW refers to psychological, physical, and social well-being [49]. In this study, we focused on EW as a dimension of psychological well-being related to satisfaction with jobs and life [50]. According to Diener [51], psychological well-being represents optimal human functioning. We conceptualized EW as the overall quality of employees’ feelings, experiences, and functioning at work [33]. Scanlan et al. [16] demonstrated that those who have experienced poor well-being at work tend to leave their jobs, and that EW has a significant negative association with turnover intention. Employees who have achieved a state of well-being in the workplace exhibit higher levels of productivity, thereby making a more substantial contribution towards organizational objectives, while also displaying reduced proclivity towards voluntary turnover [52]. Aboobaker et al. [53] also highlighted that a decline in individual well-being has been observed to contribute to a decrease in work-related outcomes such as increased rates of absenteeism, job turnover, and reduced motivation. Hence, we propose the following hypothesis:

H8 EW positively influences RI

#### 2.2.5 Mediating effect of EW

In this study, we argue that EW mediates the relationships among five core job characteristics, WR, WC, and RI. In other words, work-related factors influence RI by fostering EW. Satisfaction with the physical environment enhances EW. Previous research has demonstrated that employees who are satisfied with their physical working environment tend to have higher levels of job satisfaction, work performance, and psychological well-being [54]. Furthermore, the five core job characteristics were meaningful predictors of employees' intentions to stay [32]. Additionally, there is an extensive body of research linking WRs and WCs to RI [41,47]. Previous studies have demonstrated the motivational process of EW in the relationship between job resources and organizational outcomes (e.g., RI and perceived health) [20,55]. Employee well-being was a significant predictor of RI. Employees who are satisfied and committed tend to stay with their organization [16,56]. The JD-R model posits that EW is fostered by job resources because it plays an intrinsic motivational role by stimulating employees' growth, learning, and development and an extrinsic motivational role by providing a conducive environment for achieving work goals [23]. According to Wan et al. [22], intrinsic motivation is generated by five core job characteristics, and extrinsic motivation requires a supportive work environment. Accordingly, job resources, supportive WRs and WCs, and the five motivating job characteristics can lead to positive work outcomes. The JD-R model supports the mechanisms connecting WRs, WCs, JCs, and RI. Meaningful work indirectly affected RI by mediating the role of well-being at work [53]. Based on the above, it is plausible to infer that job resources can lead to employees' RI through EW's mediating role. Hence, we propose the following hypotheses:

H_m1-7_: EW mediates the relationship between JA, SV, TI, TS, FB, WRs, and WCs on RI

**Fig. 1:**
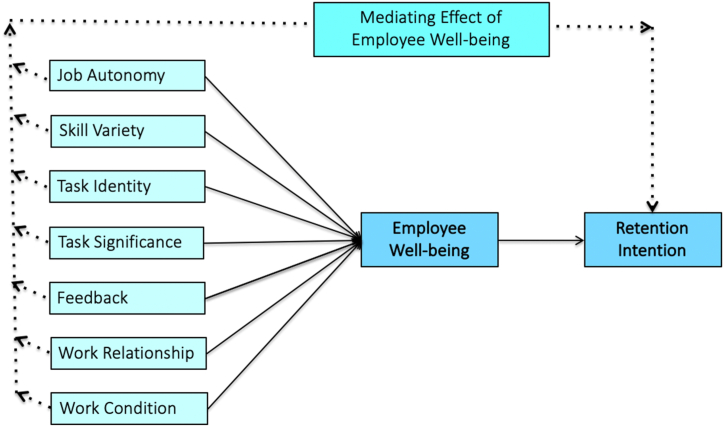
Research framework.

All associations hypothesized are presented in Fig. 1.

## 3 Research methodology

### 3.1 Data collection

This study used a quantitative approach to test the associations between variables. Rahi [57] noted that primary data are the most influential techniques in quantitative methods. Therefore, data were collected using a cross-sectional design. G*Power 3.1 was used to calculate the minimum sample size required to achieve the target analysis level and the desired minimum sample size with a power of 0.95 and an effect size of 0.15, considering the eight predictors in this study. The results indicated that at least 160 valid samples were required [58]. Nevertheless, it is recommended to use at least 200 sample sizes for partial least squares structural equation modeling (PLS-SEM) analysis [59]. The questionnaire was distributed through WJX (http://www.wjx.cn/), a widely used and practical online questionnaire management tool in China. Online data collection was conducted between August 16, 2022, and September 10, 2022. To ensure that all the participants were Chinese adults who were employed and aligned with the background of this study, judgmental questions were used for exclusion. Prior to the formal questionnaire, all participants signed an informed consent form in which they were informed of the purpose of data collection, the final destination of the data, and their right to withdraw from participation at any time. In addition, to prevent possible problems with small sample sizes, this study collected data from a relatively larger sample than the calculated minimum sample size [60]. After screening for judgmental questions and obtaining informed consent signatures, this study resulted in 804 complete responses.

### 3.2 Instrument

Overall, the scale for this study was developed based on previous literature, and the questionnaire was designed using simple, unbiased wordings so that the respondents could easily understand the questions. The English version of the items used is presented in supporting material S1, Survey Instrument. Five questions were used to measure JA as adopted from Morgeson and Humphrey [43]. The SV was measured using five items adopted from Morgeson and Humphrey [43] and Chen et al. [61]. The TI was measured using five items adopted from Morgeson and Humphrey [43] and Sims et al. [62]. The five items used to measure the TS were adopted from Morgeson and Humphrey [43] and Hackman and Oldham [32]. Five questions adopted from Morgeson and Humphrey [43] were used to measure variables including FB. WR was measured using five items adopted from Morgeson and Humphrey [43]. Five questions were used to measure variables, including EW, as adopted from Morgeson and Humphrey [43]. RI was measured using four items adopted from Diener [51]. The final scale was developed in English and translated into Chinese. To determine the correctness and validity of the questionnaire items and guarantee equivalence of the measures in the English and Chinese versions of the questionnaires, two authorized experts evaluated the final version of the developed scale. Respondents rated all variables on a five-point Likert scale (1–5, from “strongly disagree” to “strongly agree”).

### 3.3 Common method variance (CMV)

First, to investigate CMV issues, we used Harman's single-factor test in which one fixed factor was extracted from all principal constructs that explained less than 50% of the variance [63]. Single factors accounted for 33.38% of the variance, indicating that the CMV was not significant in the current study. CMV was evaluated using the full collinearity test as recommended by Kock [64]. All the variables in this study were regressed on a newly created variable from the study constructs. The variance inflation factor (VIF) for regression analysis was assessed, and the VIF scores for all input variables had to be less than 3.3 to rule out the presence of CMV. The full collinearity test results indicated the absence of CMV in the current study, as the VIF values for JA (1.446), SV (1.435), TI (1.412), TS (1.420), FB (1.381), WR (1.412), WC (1.442), EW (1.401), and RI (2.335) were less than 3.3 [64].

### 3.4 Data analysis method

Checking the multivariate normality of the data is crucial for selecting appropriate data analysis methods [65]. In this study, the multivariate normality of the data was evaluated using an online Web Power tool before conducting data analysis using SmartPLS 4.0. The tool assessed Mardia's multivariate skewness, kurtosis coefficients, and *p*-values. The results showed that the *p*-values of Mardia's multivariate skewness and kurtosis were both <0.05, indicating non-normality of the data. Subsequently, this study used PLS-SEM with SmartPLS 4.0, to test the measurement and structural models. First, bootstrapping was conducted for the measurement model to assess the reliability and validity of the research model, as well as the goodness of fit and variance inflation factor between variables. Blindfolding was then conducted to test the structural model that examined the relationship between the exogenous and endogenous latent variables [66].

## 4 Results

Table 1 presents the demographic profiles of the respondents, of whom 51.2% were male and the remainder were female. In terms of age, 47.6% of the respondents belonged to the 23–25 age group, 25.7% and 19.5% were in the age ranges of 26–30 and 18–22, respectively, and the remaining 7.1% were aged between 31 and 35. Furthermore, the average monthly income (reported as CNY: Chinese Yuan) of most respondents (44.9%) was between CNY 5000 and 8000, followed by CNY 8001 and 11000 (37.6%), CNY 11001 and 14000 (9%), less than CNY 5000 (8.2%), and higher than CNY 14000 (0.4%). Regarding annual paid leave, the majority (45.5%) of the respondents had between 11 and 20 days of annual paid leave, and only 7.6% had more than 30 days of annual paid leave. In terms of occupational sector, 25% of the respondents worked at construction firms, 20.8% and 20% worked at media firms and IT firms, respectively; 14.7% worked in the finance industry; and only 1.7% worked in other industries.

**Table 1:** Demographic details.

	N	%
*Gender*		
Male	412	51.2
Female	392	48.8
Total	804	100.0
*Age Group*		
18–22 years	157	19.5
23–25 years	383	47.6
26–30 years	207	25.7
31–35 years	57	7.1
Total	804	100.0
*Average Monthly Income (CNY)*		
Less than CNY5000	66	8.2
CNY5000–CNY8000	361	44.9
CNY8001–CNY11000	302	37.6
CNY11001–CNY14000	72	9.0
More than CNY14000	3	0.4
Total	804	100.0
*Work Experience*		
Less than 3 years	587	73.0
3–5 years	201	25.0
6–10 years	14	1.7
11–17 years	2	0.2
Total	804	100.0
*Annual Paid Leave*		
Less than 10 days	216	26.9
11–20 days	366	45.5
21–30 days	161	20.0
More than 30 days	61	7.6
Total	804	100.0
*Education*		
Secondary school certificate	65	8.1
Diploma certificate	268	33.3
Bachelor's degree or equivalent	365	45.4
Master's degree	94	11.7
Doctoral degree	12	1.5
Total	804	100.0
*Marital Status*		
Single	188	23.4
Married	595	74.0
Divorced	17	2.1
Widowed	4	0.5
Total	804	100.0
*Types of Organization*		
IT Firms	161	20.0
Finance	118	14.7
Construction	201	25.0
Media	167	20.8
Education	143	17.8
Others	14	1.7
Total	804	100.0
*Yearly Bonus*		
Less than CNY10000	245	30.5
CNY10001–CNY30000	404	50.2
CNY30001–CNY50000	103	12.8
More than CNY50000	52	6.5
Total	804	100.0

### 4.1 Reliability and validity

**Fig. 2:**
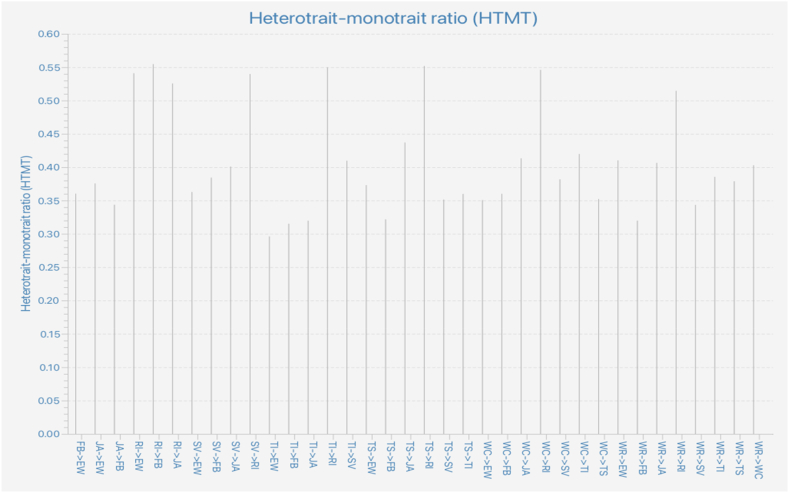
Heterotrait-monotrait ratio.

Hair et al. [59] recommended that before measuring the structural model, it is necessary to ensure the reliability and validity of the measurement model. Table 2 shows the bootstrapping results and mean and standard deviation for each construct. The reflective measurement models were evaluated using internal consistency, convergent validity, and discriminant validity [66]. Cronbach's alpha, composite reliability, and Dijkstra–Hensel's rho were used to evaluate the internal consistency. The results in Table 2 show that Cronbach's alpha, composite reliability, and Dijkstra's rho were all greater than the recommended threshold of 0.7, indicating the construct's good internal consistency. The loading values were all greater than 0.7, and the average variance extracted (AVE) values ranged from 0.726 to 0.706, which were greater than the recommended threshold of 0.5, indicating good convergent validity of the construct. Additionally, all VIFs were less than 3.3, indicating no serious collinearity issues within the construct [64]. Finally, Fig. 2 shows that the Heterotrait-Monotrait Ratio was less than 0.55, indicating good discriminant validity between the constructs in this study [59]. The loading and cross-loading values are presented in supporting materials ***S2:*** *Discriminant Validity* showed that all loadings were more than 0.5, which is higher than the respective cross-loading values. These results suggest that the construct is suitable for blindfolding and evaluating the structural model in the next step.

**Table 2:** Reliability and validity.

Variables	No. of Items	Mean	Standard Deviation	Cronbach's Alpha	Dijkstra-Hensele's *rho*	Composite Reliability	Average Variance Extracted	Variance Inflation Factors
JA	5	3.686	0.922	0.898	0.903	0.925	0.711	1.409
SV	5	3.544	0.972	0.902	0.905	0.927	0.719	1.370
TI	5	3.726	0.913	0.899	0.900	0.925	0.712	1.348
TS	5	3.692	0.921	0.899	0.901	0.925	0.713	1.336
FB	5	3.397	1.007	0.901	0.902	0.927	0.717	1.268
WR	5	3.837	0.889	0.896	0.898	0.923	0.706	1.348
WC	5	3.877	0.890	0.898	0.905	0.924	0.710	1.392
EW	5	3.645	0.936	0.899	0.899	0.925	0.713	1.000
RI	4	3.660	0.921	0.874	0.875	0.914	0.726	–

**Note:** JA - Job Autonomy, SV - Skill Variety, TI - Task Identity, TS - Task Significance, FB - Feedback, WR - Work Relationship, WC - Work Condition, EW - Employee Well-being, RI - Retention Intention.

### 4.2 Hypotheses testing

Verifying the reliability and validity of the outer model allowed us to evaluate the inner-path model estimates. We calculated the coefficients of determination (*R*^*2*^) and effect sizes (*f*^*2*^). The *R*^*2*^ value represents the variance in the dependent variable as explained by the independent variables. In other words, it is the proportion of variability in the data explained by the measurement model. The values of *R*^*2*^ ranged from 0 to 1, with a higher value indicating that the model had greater explanatory power. As shown in Table 3, the *R*^*2*^ value was 0.257 for EW, indicating that 25.7% of the EW variation could be explained by JA, SV, TI, TS, TS, FB, WR, and WC. This moderate *R*^*2*^ value was considered acceptable because this study was not designed to identify which factors generally affect EW and RI; rather, it attempted to identify how JA, SV, TI, TS, TS, FB, WR, and WC affect EW and RI in the context of work design. In terms of RI, the *R*^*2*^ value was 0.231, indicating that EW explained 23.1% of the variation in turnover intention.

**Table 3:** Hypothesis testing.

Hypothesis		Beta	CI Min	CI Max	*t* *Value*	*p* *value*	*R*^2^	*f*^*2*^	Decision
H_1_	JA→EW	0.105	0.036	0.176	2.485	0.006		0.011	Supported
H_2_	SV→EW	0.111	0.042	0.179	2.682	0.004		0.012	Supported
H_3_	TI→EW	0.023	−0.044	0.095	0.554	0.290		0.001	Rejected
H_4_	TS→EW	0.127	0.058	0.195	3.095	0.001	0.257	0.016	Supported
H_5_	FB→EW	0.133	0.067	0.197	3.373	0.000		0.019	Supported
H_6_	WR→EW	0.176	0.110	0.244	4.300	0.000		0.031	Supported
H_7_	WC→EW	0.085	0.019	0.155	2.051	0.020		0.007	Supported
H_8_	EW→RI	0.480	0.430	0.529	15.822	0.000	0.231	0.300	Supported
H_M1_	JA→EW→RI	0.051	0.017	0.086	2.412	0.008			Supported
H_M2_	SV→EW→RI	0.054	0.020	0.087	2.617	0.004			Supported
H_M3_	TI→EW→RI	0.011	−0.021	0.046	0.551	0.291			Rejected
H_M4_	TS→EW→RI	0.061	0.028	0.095	2.992	0.001			Supported
H_M5_	FB→EW→RI	0.064	0.031	0.098	3.173	0.001			Supported
H_M6_	WR→EW→RI	0.084	0.052	0.120	4.087	0.000			Supported
H_M7_	WC→EW→RI	0.041	0.009	0.075	2.006	0.022			Supported

**Note:** JA - Job Autonomy, SV - Skill Variety, TI - Task Identity, TS - Task Significance, FB - Feedback, WR - Work Relationship, WC -Work Condition, EW - Employee Well-being, RI - Retention Intention.

**Fig. 3:**
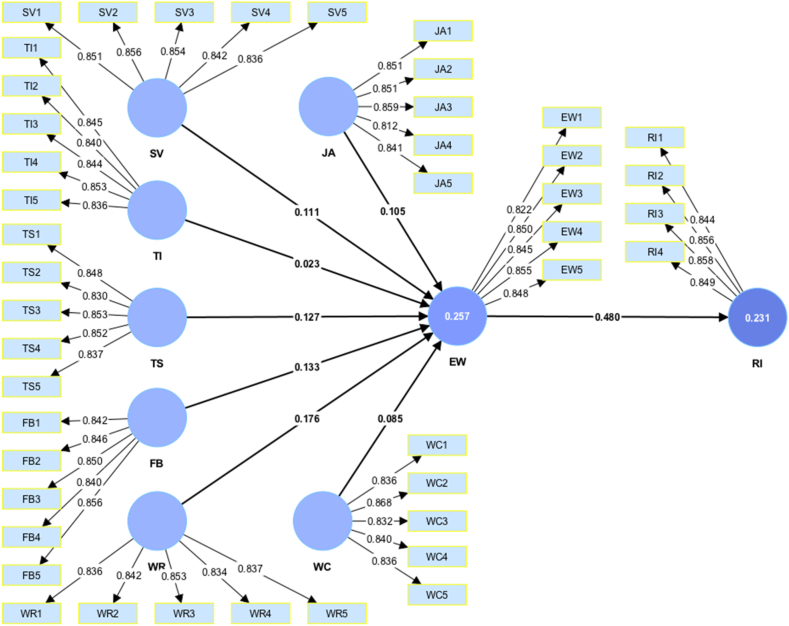
Measurement model.

As shown in Table 3 and Fig. 3, the path coefficient of JA on EW achieved an acceptable *p*-value, which provided evidence supporting H_1_. The path value of SV on EW was also statistically significant; therefore, H_2_ was supported. However, the path value of TI on EW was not significant; hence, H_3_ was rejected. The path values between TS and EW, FB and EW, WR and EW, and WC and EC were positive and significant. Hence, H_4_, H_5_, H_6_, and H_7_ were supported, and the path value between EW on RI was also positive and statistically significant, thus supporting H_8_.

### 4.3 Mediation analysis

The findings of the mediation study presented in Table 3 show that EW was a significant mediator in the paths from JA, SV, TS, FB, WR, and WCs to RI. This result supports H_M1_, H_M2_, H_M4_, H_M5_, H_M6_, and H_M7_. However, EW's mediating role in the relationship between work identification and RI was insignificant. Hence, H_M3_ was rejected.

### 4.4 Importance–performance matrix analysis

**Fig. 4:**
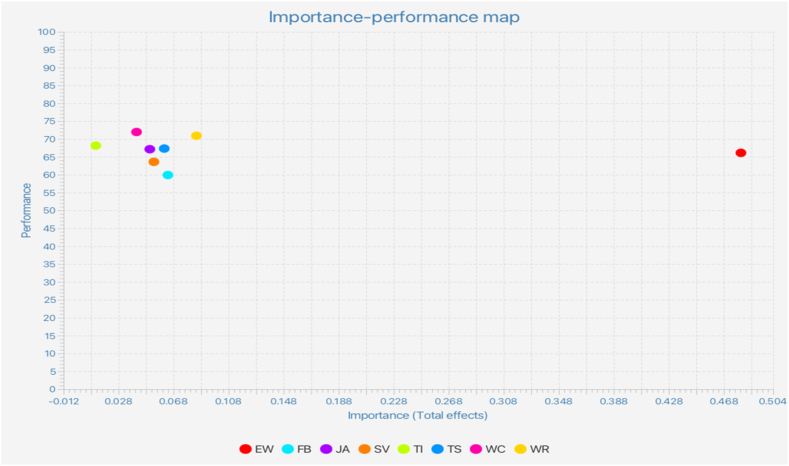
Importance-performance map analysis.

To further examine the results, this study conducted an importance-performance matrix analysis (IPMA) using JA, SV, TI, TS, FB, WRs, WCs, and EW as variables and RI as the target construct. The objective was to identify the input variables that are important despite their low performance and thus have a strong overall effect on the construct. Consequently, by focusing on poorly performing, but crucial variables, an IPMA can provide detailed information on the most effective managerial actions. As shown in Fig. 4, EW had the highest importance and highest total effect on RI. Compared to the other variables, the EW performance was slightly below average. Hence, when managers aim to increase the performance of RI (i.e., the target construct), their first priority should be to improve the performance of aspects captured by EW, as this construct has the highest importance but relatively low performance. Aspects related to WR should be prioritized.

## 5 Discussion and implications

This study investigated the impact of five core job characteristics on the well-being of young Chinese employees. Consistent with our hypothesis (H_1_), the findings revealed that job autonomy positively influenced employee well-being. This result is in line with previous research conducted by Clausen et al. [67], who demonstrated that job autonomy is positively associated with psychological well-being. This indicates that higher levels of job autonomy are beneficial for employee well-being. In addition, the results of this study show a positive effect of skill variety on EW. Thus, H_2_ is supported, which supports the findings of prior studies on employees’ work engagement [68,69]. These studies highlighted that skill variety is most predictive of work engagement when job demands were high. This study builds on previous research and provides additional practical and theoretical significance regarding the influence of skill variety on employee well-being. However, contrary to H_3_, we found no positive relationship between task identity and employee well-being. This finding conflicts with a recent study by Jiang et al. [70], in which task identity was examined as a significant positive factor affecting thriving at work and subsequently EW. This discrepancy may be due to differences in the measures used to assess task identity or demographic factors as young Chinese workers may have unique work values [71]. Rather than focusing on the specific tasks or responsibilities of their jobs, young Chinese workers may be more concerned with the overall social and cultural context of their work environments. Future studies should explore this relationship in other contexts.

Furthermore, consistent with H_4_, the results indicated a significant and positive relationship between task significance and employee well-being. This finding is in line with those of previous studies conducted by Zhu et al. [72] and Grant [73], which indicated that individuals who perceive their work as significant are more likely to experience greater meaningfulness in their work, leading to improved well-being. In addition, this study examines whether the relationship between feedback and EW is significant and positive, thus supporting H_5_. This finding is consistent with previous research by Johari et al. [34]. These authors suggested that feedback is critical for eliciting favorable workplace outcomes and improving employee well-being. Moreover, studies conducted by Radic et al. [74] and Bakker [75] supported our findings by demonstrating that feedback loops positively influence work engagement and well-being. These findings reinforce the importance of feedback as a critical predictor of wellbeing among young employees.

The results also reported a significant positive impact of WR on EW (H_6_), which supports the findings of prior studies [40,76]. The findings indicate that when young Chinese workers work in harmonious workplaces and have good relationships with colleagues and peers, their employee satisfaction and EW improve. A company's employees share working relationships and build expectations for the conduct of others regarding a particular task based on the information accessible to them. Once these expectations are fulfilled, employees feel more connected and satisfied at work, which improves their mental health and performance [41,77]. Additionally, this study empirically demonstrated the significant influence of WC on EW (H_7_). Edwards et al. [45], Morgeson and Humphrey [43], and Ravalier et al. [48] reported similar findings regarding the significant influence of WC on EW. This study adds to the existing literature on the importance of job resources such as WRs and WCs in improving employee well-being, which has implications for organizations seeking to improve their employees' well-being and performance.

Furthermore, this study sheds light on the relationship between EW and RI among young Chinese workers. Specifically, this study revealed that employees’ emotional well-being positively influences their RIs (H_8_). Prior studies have typically focused on the negative association between employee well-being and turnover intention [16,52]. In contrast, our study highlights the positive association between employee well-being and RI, suggesting that promoting well-being may be an effective means of retaining young Chinese workers. The current findings suggest that employees who experience high levels of well-being in the workplace are more likely to have a higher level of RI towards their current workplace, which has important implications for organizations seeking to retain their employees and underscores the importance of promoting employee well-being as a means of achieving this goal.

The results of the mediation analysis confirmed that four of the five core job characteristics (i.e., job autonomy, skill variety, task significance, and feedback) had an indirect effect on RI mediated by EW (H_M1_, H_M2_, H_M4_, and H_M5_). These findings are consistent with those of Schaufeli and Taris [20], who presented a revised JD-R model and demonstrated that job resources, such as motivational job characteristics, social support, and safety climate, indirectly influence work outcomes (e.g., turnover intention) through the mediating effect of well-being. Hence, these findings contribute to the JCM model by introducing a new psychological state (i.e., well-being) that is influenced by job characteristics and, in turn, influences job attitudes, such as RI. The mediation results obtained in this study also confirm that EW is a significant mediator of the relationship between WR and RI (H_M6_), which supports the findings of several previous studies [78,79]. Additionally, the findings suggest that EW mediates the relationship between WC and RI (H_M7_), consistent with McGuire and McLaren [54], which confirmed that employee well-being mediates the relationship between the physical work environment and employee commitment. Hence, the significance of well-being as a mediator in the relationships between WR, WC, and job attitudes should not be overlooked. However, the mediating roles of task identity and RI are not significant (H_M3_). This may be attributed to the cultural values of Chinese workers, who prioritize collectivism and cooperation in their work [80] and may not attach great importance to their task identity. Future researchers could test this model in other countries that advocate for an individualistic culture among workers.

### 5.1 Practical implications

The current study's findings offer imperative practical inferences for organizations concerning the retention of young and talented employees. This study demonstrates that young workers' perceptions of motivational job characteristics are a tool for enhancing their RI. These findings indicate that improving aspects related to work design affect workers' well-being, thus promoting RI among young Chinese workers, although there is the possibility of individual differences in work design. Hence, organizations should formulate strategies in the context of work design to increase the RI of young workers. For example, managers could redesign job roles to incorporate motivational characteristics that create a more meaningful, challenging, and engaging work environment for employees. One way to achieve this is to develop a performance management system that provides regular and constructive feedback to employees regarding their work, thereby enabling them to identify areas for improvement and achieve their goals more effectively. Additionally, organizations can offer training programs, workshops, and mentorship opportunities to help employees improve their skills, expand their knowledge, and advance their careers, as young workers have higher expectations of career development than their older co-workers [3].

Based on the finding that there is a positive relationship between WR and RI, it is advisable for organizations to prioritize the development of positive WR among young workers. This can be achieved by promoting teamwork, organizing social events, and fostering a positive work culture. Moreover, investing in training programs that improve communication skills and encourage collaboration can significantly enhance the quality of WR. Training programs can help employees develop a better understanding of different communication styles and preferences, facilitating more effective communication, and reducing misunderstandings and conflicts. By learning how to communicate more clearly and efficiently, employees can avoid unnecessary misunderstandings and build trust and rapport with their coworkers. Therefore, investing in initiatives that foster positive WRs may be an effective strategy for preserving RIs and maintaining a committed and engaged workforce.

The current research has also shown that the creation of a supportive and positive WC that prioritizes the well-being of employees can increase young workers' RI. Therefore, organizations should prioritize the improvement of their employees’ physical and psychological WC. This can be achieved by implementing ergonomic workstations, providing proper lighting, and ensuring comfortable temperatures. Furthermore, organizations should establish policies and practices that promote a positive work-life balance, such as flexible work schedules and parental leave. By creating a supportive work environment that prioritizes employee well-being, organizations can foster a sense of commitment [81], which in turn can lead to increased RI and decreased employee turnover.

### 5.2 Theoretical implications

The findings of the current study have several important implications for theory and the existing literature. First, within the RI literature, this study reaffirms the role of motivating job characteristics, WR, and WC, in enhancing employee RI [22,56]. By shedding light on the specific processes and configurations that contribute to RI, this research also responds to calls for a more comprehensive understanding of the impact of work design [43,68]. Specifically, the findings highlight the critical role of job characteristics that provide employees with a sense of autonomy, skill variety, task significance, and feedback to enhance their RI. Furthermore, positive WRs, characterized by social support and meaningful interactions with supervisors and coworkers, were key factors in promoting RI. Favorable WCs, such as comfortable temperatures, tidiness in the workplace, and job security, were also identified as important factors contributing to RI. Additionally, this study extends the job characteristics model proposed by Hackman and Oldham [27] by considering a new psychological state, employee well-being, which is influenced by job characteristics. The JCM model posits that five job characteristics enhance job attitudes by promoting three critical psychological states, such as an experience of job meaningfulness, a sense of responsibility for work outcomes, and knowledge of work results. By considering EW as an additional psychological state, this study advances our understanding of how job characteristics influence RIs among young employees.

## 6 Conclusion

This study demonstrates that motivational job characteristics, WRs, and conditions have a significant impact on RI through employee well-being. Job autonomy, skill variety, task significance, and feedback are crucial predictors of both employee well-being and RI. Furthermore, positive WRs among colleagues and a comfortable work environment can enhance employee well-being, leading to higher RI. Employees who experience higher levels of well-being are more likely to remain in their current roles. Therefore, incorporating motivational job characteristics into job design, and improving WCs and relationships can enhance employee well-being and RI. This, in turn, promotes employee retention and contributes to the achievement of organizational goals.

### 6.1 Limitations and future research directions

This study has some limitations that should be considered when interpreting the results and drawing their implications. First, the results have limited generalizability. The sample size of this study was small and was limited to the Chinese population. Although the findings of this study are largely consistent with those of previous studies conducted in more diverse contexts, we recommend that our study be replicated in other regions and sectors to better understand the nature of our findings. Second, because this study used a cross-sectional design, causal relationships among the study variables could not be ascertained. Future studies should use longitudinal data collection methods. Third, while this study examined EW as a mediator, future studies may consider examining other mediating factor(s), such as work engagement and job satisfaction. Future research could attempt to integrate more constructs related to work design into the study model to reveal a deeper and more generalized understanding.

## Declarations

### Author(s) contribution

*Chen Xuelin*: Conceived and designed the experiments; Performed the experiments; Wrote the paper. *Abdullah Al Mamun*: Performed the experiments; Analyzed and interpreted the data; Contributed reagents, materials, analysis tools or data; Wrote the paper. *Mohammad Enamul Hoque*: Conceived and designed the experiments; Performed the experiments; Wrote the paper. *Wan Mohd Hirwani Wan Hussain*: Conceived and designed the experiments; Performed the experiments; Wrote the paper. *Qing Yang*: Conceived and designed the experiments; Analyzed and interpreted the data; Wrote the paper. All authors approved the final version of the manuscript and give their consent for submission and publication.

### Availability of data and materials

The original contributions presented in the study are included in the article/Supplementary Material, further inquiries can be directed to the corresponding author/s.

### Conflict of interest

The authors declare that the research was conducted in the absence of any commercial or financial relationships that could be construed as a potential conflict of interest.

### Funding

This research received no specific grant from any funding agency in the public, commercial, or not-for-profit sectors.
