# Supplementary material for: Work design, employee well-being, and retention intention: A case study of China's young workforce
Source: Heliyon. 2023 Apr 24;9(5):e15742. doi: 10.1016/j.heliyon.2023.e15742 (PMC10163660; doi:10.1016/j.heliyon.2023.e15742)
Supplement: Multimedia component 1 [file mmc1.docx]

**S1.** Survey Questionnaire

| Code | Items |
| --- | --- |
| JA1 | The job allows me to make my own decisions about how to schedule my work. |
| JA2 | The job allows me to decide on the order in which things are done on the job. |
| JA3 | The job allows me to make a lot of decisions on my own |
| JA4 | The job allows me to use my personal initiative or judgment in carrying out the work |
| JA5 | The job allows me to make decisions about what methods I use to complete my work |
| SV1 | The job requires a variety of skills. |
| SV2 | The job requires me to utilize a variety of different skills in order to complete the work. |
| SV3 | The job requires me to use a number of complex or high-level skills. |
| SV4 | The job requires the use of a number of skills. |
| SV5 | The job requires me to possess various skills or knowledge through training. |
| TI1 | The job involves completing a piece of work that has an obvious beginning and end |
| TI2 | The job is arranged so that I can do an entire piece of work from beginning to end |
| TI3 | The job provides me the chance to completely finish the pieces of work I begin. |
| TI4 | The job allows me to complete work I start. |
| TI5 | The job offers me the opportunity to do a whole job. |
| TS1 | The results of my work are likely to significantly affect the lives of other people. |
| TS2 | The job itself is very significant and important in the broader scheme of things. |
| TS3 | The job has a large impact on people outside the organization. |
| TS4 | The work performed on the job has a significant impact on people outside the organization. |
| TS5 | A lot of people can be positively affected by how well my job gets done |
| FB1 | I receive feedback on my performance from the job itself. |
| FB2 | I receive direct and clear information about the effectiveness (e.g., quality and quantity) of my job performance from the work activities themselves. |
| FB3 | I receive a great deal of information from my manager and coworkers about my job performance. |
| FB4 | I receive information about the effectiveness of my job performance from other people in the organization, such as managers and coworkers |
| FB5 | I receive feedback on my performance from other people in my organization (such as my manager or coworkers). |
| WR1 | I have the opportunity to develop close friendships in my job |
| WR2 | I have the chance in my job to get to know other people |
| WR3 | My supervisor is concerned about the welfare of the people that work for him/her. |
| WR4 | People I work with take a personal interest in me. |
| WR5 | People I work with are friendly |
| WC1 | The work place is free from excessive noise. |
| WC2 | The climate at the work place is comfortable in terms of temperature and humidity. |
| WC3 | The job has a low risk of accident. |
| WC4 | The job takes place in an environment free from health hazards (e.g., chemicals, fumes, etc.). |
| WC5 | The job occurs in a clean environment. |
| EW1 | I am living in a purposeful and meaningful life. |
| EW2 | I am engaged and interested in my daily activities. |
| EW3 | I am competent and capable in the activities that are important to me. |
| EW4 | I am a good person and live a good life. |
| EW5 | I am optimistic about my future. |
| RI1 | I like the organization where I currently work |
| RI2 | I am willing to work hard for the development of this organization |
| RI3 | I do not envy my colleagues who have left this organization |
| RI4 | In the past 6 months, I have not looked for another job. |

**Note:** JA - Job autonomy, SV - Skill Variety, TI - Task Identity, TS - Task Significance, FB – Feedback, WR - Work Relationships, WC - Work Environment, EW - Employee Wellbeing, RI - Retention Intention

**S2.** Discriminant Validity

| Code | JA | SV | TI | TS | FB | WR | WC | EW | RI |
| --- | --- | --- | --- | --- | --- | --- | --- | --- | --- |
| JA1 | 0.851 | 0.287 | 0.244 | 0.305 | 0.244 | 0.325 | 0.324 | 0.312 | 0.403 |
| JA2 | 0.851 | 0.340 | 0.278 | 0.373 | 0.284 | 0.352 | 0.317 | 0.308 | 0.410 |
| JA3 | 0.859 | 0.322 | 0.233 | 0.333 | 0.297 | 0.332 | 0.355 | 0.295 | 0.407 |
| JA4 | 0.812 | 0.267 | 0.236 | 0.317 | 0.235 | 0.258 | 0.279 | 0.251 | 0.378 |
| JA5 | 0.841 | 0.307 | 0.222 | 0.326 | 0.243 | 0.273 | 0.299 | 0.258 | 0.366 |
| SV1 | 0.284 | 0.851 | 0.358 | 0.257 | 0.298 | 0.263 | 0.291 | 0.267 | 0.409 |
| SV2 | 0.323 | 0.856 | 0.296 | 0.263 | 0.281 | 0.285 | 0.322 | 0.283 | 0.415 |
| SV3 | 0.303 | 0.854 | 0.313 | 0.293 | 0.324 | 0.260 | 0.288 | 0.310 | 0.407 |
| SV4 | 0.314 | 0.842 | 0.305 | 0.266 | 0.307 | 0.285 | 0.310 | 0.277 | 0.406 |
| SV5 | 0.311 | 0.836 | 0.294 | 0.264 | 0.259 | 0.217 | 0.249 | 0.249 | 0.397 |
| TI1 | 0.222 | 0.304 | 0.845 | 0.273 | 0.249 | 0.278 | 0.318 | 0.219 | 0.419 |
| TI2 | 0.238 | 0.294 | 0.840 | 0.280 | 0.242 | 0.282 | 0.323 | 0.225 | 0.417 |
| TI3 | 0.262 | 0.303 | 0.844 | 0.313 | 0.234 | 0.307 | 0.307 | 0.236 | 0.414 |
| TI4 | 0.251 | 0.343 | 0.853 | 0.251 | 0.252 | 0.302 | 0.316 | 0.236 | 0.406 |
| TI5 | 0.243 | 0.313 | 0.836 | 0.251 | 0.218 | 0.290 | 0.322 | 0.207 | 0.400 |
| TS1 | 0.320 | 0.254 | 0.271 | 0.848 | 0.253 | 0.239 | 0.266 | 0.290 | 0.418 |
| TS2 | 0.313 | 0.248 | 0.245 | 0.830 | 0.221 | 0.258 | 0.254 | 0.263 | 0.418 |
| TS3 | 0.362 | 0.270 | 0.280 | 0.853 | 0.221 | 0.306 | 0.268 | 0.292 | 0.395 |
| TS4 | 0.371 | 0.290 | 0.286 | 0.852 | 0.285 | 0.354 | 0.281 | 0.273 | 0.438 |
| TS5 | 0.293 | 0.278 | 0.286 | 0.837 | 0.242 | 0.277 | 0.266 | 0.300 | 0.398 |
| FB1 | 0.265 | 0.282 | 0.216 | 0.268 | 0.842 | 0.219 | 0.293 | 0.272 | 0.416 |
| FB2 | 0.234 | 0.277 | 0.233 | 0.212 | 0.846 | 0.246 | 0.265 | 0.268 | 0.398 |
| FB3 | 0.268 | 0.318 | 0.262 | 0.231 | 0.850 | 0.253 | 0.277 | 0.267 | 0.432 |
| FB4 | 0.307 | 0.317 | 0.262 | 0.287 | 0.840 | 0.226 | 0.285 | 0.272 | 0.437 |
| FB5 | 0.241 | 0.280 | 0.230 | 0.229 | 0.856 | 0.272 | 0.248 | 0.297 | 0.404 |
| WR1 | 0.276 | 0.259 | 0.278 | 0.272 | 0.245 | 0.836 | 0.295 | 0.284 | 0.371 |
| WR2 | 0.321 | 0.267 | 0.297 | 0.294 | 0.235 | 0.842 | 0.311 | 0.316 | 0.407 |
| WR3 | 0.312 | 0.265 | 0.268 | 0.246 | 0.248 | 0.853 | 0.317 | 0.336 | 0.384 |
| WR4 | 0.291 | 0.250 | 0.282 | 0.279 | 0.248 | 0.834 | 0.280 | 0.305 | 0.359 |
| WR5 | 0.347 | 0.262 | 0.330 | 0.338 | 0.234 | 0.837 | 0.320 | 0.308 | 0.393 |
| WC1 | 0.313 | 0.295 | 0.346 | 0.265 | 0.286 | 0.312 | 0.836 | 0.241 | 0.423 |
| WC2 | 0.340 | 0.314 | 0.322 | 0.265 | 0.280 | 0.321 | 0.868 | 0.309 | 0.419 |
| WC3 | 0.332 | 0.277 | 0.290 | 0.277 | 0.247 | 0.319 | 0.832 | 0.286 | 0.386 |
| WC4 | 0.300 | 0.288 | 0.307 | 0.242 | 0.266 | 0.282 | 0.840 | 0.262 | 0.393 |
| WC5 | 0.286 | 0.279 | 0.323 | 0.286 | 0.285 | 0.292 | 0.836 | 0.230 | 0.416 |
| EW1 | 0.280 | 0.319 | 0.246 | 0.282 | 0.276 | 0.303 | 0.271 | 0.822 | 0.412 |
| EW2 | 0.267 | 0.278 | 0.210 | 0.269 | 0.247 | 0.326 | 0.271 | 0.850 | 0.407 |
| EW3 | 0.291 | 0.258 | 0.216 | 0.278 | 0.292 | 0.335 | 0.257 | 0.845 | 0.398 |
| EW4 | 0.303 | 0.264 | 0.215 | 0.297 | 0.309 | 0.309 | 0.274 | 0.855 | 0.419 |
| EW5 | 0.295 | 0.267 | 0.239 | 0.294 | 0.248 | 0.285 | 0.271 | 0.848 | 0.388 |
| RI1 | 0.374 | 0.408 | 0.411 | 0.391 | 0.390 | 0.407 | 0.415 | 0.408 | 0.844 |
| RI2 | 0.392 | 0.408 | 0.425 | 0.417 | 0.434 | 0.381 | 0.400 | 0.417 | 0.856 |
| RI3 | 0.416 | 0.415 | 0.401 | 0.450 | 0.444 | 0.385 | 0.395 | 0.418 | 0.858 |
| RI4 | 0.410 | 0.402 | 0.423 | 0.408 | 0.409 | 0.380 | 0.437 | 0.393 | 0.849 |
| *Fornell-Larcker criterion* | | | |  |  |  |  |  |  |
| JA | 0.843 |  |  |  |  |  |  |  |  |
| SV | 0.362 | 0.848 |  |  |  |  |  |  |  |
| TI | 0.289 | 0.369 | 0.844 |  |  |  |  |  |  |
| TS | 0.393 | 0.318 | 0.325 | 0.844 |  |  |  |  |  |
| FB | 0.310 | 0.348 | 0.284 | 0.289 | 0.847 |  |  |  |  |
| WR | 0.369 | 0.310 | 0.346 | 0.339 | 0.288 | 0.840 |  |  |  |
| WC | 0.375 | 0.345 | 0.376 | 0.316 | 0.322 | 0.363 | 0.843 |  |  |
| EW | 0.340 | 0.329 | 0.267 | 0.336 | 0.326 | 0.370 | 0.319 | 0.844 |  |
| RI | 0.467 | 0.480 | 0.487 | 0.489 | 0.492 | 0.456 | 0.483 | 0.480 | 0.852 |

**Note:** JA - Job autonomy, SV - Skill Variety, TI - Task Identity, TS - Task Significance, FB – Feedback, WR - Work Relationships, WC - Work Environment, EW - Employee Wellbeing, RI - Retention Intention
